# Preventive Electroacupuncture Ameliorates D-Galactose-Induced Alzheimer's Disease-Like Pathology and Memory Deficits Probably via Inhibition of GSK3*β*/mTOR Signaling Pathway

**DOI:** 10.1155/2020/1428752

**Published:** 2020-04-23

**Authors:** Chao-Chao Yu, Jia Wang, Si-Si Ye, Shan Gao, Jia Li, Li Wang, Tao Jiang, Xue-Song Wang, Bo-Cun Li, Qing Shu, Wei Lu, Yan-Jun Du, Li-Hong Kong

**Affiliations:** ^1^College of Acupuncture and Orthopedics, Hubei University of Chinese Medicine, Wuhan, Hubei, China; ^2^Hubei Provincial Collaborative Innovation Center of Preventive Treatment by Acupuncture and Moxibustion, Hubei University of Chinese Medicine, Wuhan, Hubei, China; ^3^Fourth Clinical Medical College, Guangzhou University of Traditional Chinese Medicine, Guangzhou, China; ^4^Department of Acupuncture & Moxibustion, Wuhan Hospital of Integrated Chinese & Western Medicine, Tongji Medical College of Huazhong University of Science & Technology, Wuhan, Hubei, China; ^5^Department of Acupuncture, Union Hospital, Tongji Medical College, Huazhong University of Science and Technology, Wuhan, Hubei, China; ^6^Department of Rehabilitation, Zhongnan Hospital of Wuhan University, Wuhan University, Wuhan, Hubei, China

## Abstract

Acupuncture has been practiced to treat neuropsychiatric disorders for a thousand years in China. Prevention of disease by acupuncture and moxibustion treatment, guided by the theory of Chinese acupuncture, gradually draws growing attention nowadays and has been investigated in the role of the prevention and treatment of mental disorders such as AD. Despite its well-documented efficacy, its biological action remains greatly invalidated. Here, we sought to observe whether preventive electroacupuncture during the aging process could alleviate learning and memory deficits in D-galactose-induced aged rats. We found that preventive electroacupuncture at GV20-BL23 acupoints during aging attenuated the hippocampal loss of dendritic spines, ameliorated neuronal microtubule injuries, and increased the expressions of postsynaptic PSD95 and presynaptic SYN, two important synapse-associated proteins involved in synaptic plasticity. Furthermore, we observed an inhibition of GSK3*β*/mTOR pathway activity accompanied by a decrease in tau phosphorylation level and prompted autophagy activity induced by preventive electroacupuncture. Our results suggested that preventive electroacupuncture can prevent and alleviate memory deficits and ameliorate synapse and neuronal microtubule damage in aging rats, which was probably via the inhibition of GSK3*β*/mTOR signaling pathway. It may provide new insights for the identification of prevention strategies of AD.

## 1. Introduction

Alzheimer's disease (AD), a neurodegenerative disease accounting for nearly 80% in all dementia diseases, is characterized by progressive memory decline, executive dysfunction, personality and behavioral changes, and other neuropsychiatric syndromes [1]. It is estimated that there will probably be 132 million AD patients worldwide by 2050 [2]. The incidence of AD is closely related to aging [3]. As the worldwide population ages, AD has become one of the most important medical and social problems in the world [4]. Although China has recently approved sodium oligomannate (GV-971), a marine algae-derived oral oligosaccharide able to recondition gut microbiota and alleviate neuroinflammation [5], for the treatment of mild to moderate AD patients [6], more experimental and clinical evidence for its mechanism of action, long-term efficacy and safety in the treatment of AD is still needed. Since AD is insidious and its progression is difficult to reverse, it is of great significance to explore the early prevention strategy.

Extracellular A*β* plaques and intracellular neurofibrillary tangles (NFTs) composed of hyperphosphorylated tau proteins in the cerebral cortex and hippocampus are the typical pathological changes of AD [2, 7]. It is well recognized that the severity of dementia is strongly correlated with NFTs rather than senile plaques [8, 9]. NFTs deposition as a result of tau hyperphosphorylation is recognized as the early pathological hallmark of AD [8, 10–12]. Microtubule-associated protein tau is highly expressed in the axons of neurons in the central nervous system [13]. Its binding to microtubules can maintain the stability of microtubules, which is of great significance for maintaining normal axonal plasmic transport and synaptic plasticity [13]. Paired helical filaments (PHFs) are the major component of NFTs [14, 15]. The aggregation of hyperphosphorylated tau protein or disorder of its degradation contributes to the NFTs deposition in neurons. NFTs deposition at the dendritic spine of neurons mediates the neurotoxicity of A*β*, resulting in the loss and morphological changes of dendritic spines, which directly leads to the damage of neuronal synaptic function [13]. Given the fact that AD is one of the best known aging‐linked neurodegenerative diseases [16–18], prevention before NFTs load during aging could be providing another clue for drug development.

Glycogen synthase kinase 3*β* (GSK3*β*) is a serine/threonine protein kinase and is involved in inducing tau phosphorylation [19]. Mounting evidence indicated that inhibition of GSK3*β* significantly attenuated cognition deficits caused by tau hyperphosphorylation [19–21]. A recent study found that NFTs deposition and neuron loss were closely related to autophagy activity in neurons and were aggravated with the progression of AD [22]. Several studies showed that the negative regulator of autophagy mammalian target of rapamycin (mTOR) was the downstream target of GSK3*β* [23–25]. Inhibition of GSK3 *β* restored the acidification of autophagy lysosome via inhibiting mTOR, promoted the autophagy clearance of pathological *β*-amyloid protein, and alleviated cognitive impairment in AD mice [25]. Siman et al. [26] also found that enhanced autophagy activity prevented the entorhinal cortex and perforant pathway projection from tau-mediated neurodegeneration, synapse loss, and neuroinflammation. In addition, the GSK3*β*/mTOR pathway is involved in the regulation of glucose metabolism [27], and disordered glucose metabolism is also implicated in the pathogenesis of AD [28]. So it is reasonably assumed that downregulation on GSK3*β*/mTOR pathway to inhibit tau hyperphosphorylation and promote autophagy could be a potential approach for treating AD.

Acupuncture, a form of traditional Chinese medicine, is one of the most popular forms of complementary and alternative medicine used in western countries. It has a long history of the application of acupuncture therapy in the treatment of various neurological diseases in China. Mounting evidence from clinical trials [29, 30] and animal studies [31] indicate that acupuncture may be a potential approach for treating cognitive dysfunction in AD. In our previous studies, we also found that high-frequency electroacupuncture ameliorated synapse injury and memory impairment via inhibiting GSK3*β* in A*β*_1-42_-induced AD rats [32, 33]. It is widely recognized that aging is a significant risk factor for the development and progression of AD, and prevention strategies targeting hallmarks of aging associated with AD could be a potential approach for preventing or treating AD [3, 34, 35]. Currently, studies investigating effects of acupuncture on AD mainly focus on transgenic or A*β*-induced AD mouse, whether preventive acupuncture intervention during aging could alleviate cognitive impairments in aging animal model and possible underlying mechanisms are less studied. Herein, we sought to observe whether the synchronous electroacupuncture treatment during aging could be able to prevent or alleviate cognitive decline in aging rats.

## 2. Materials and Methods

### 2.1. Animals and Groups

Forty-eight adult male Sprague Dawley rats approximately aged three months and weighing 330 ± 20 g were obtained from the laboratory animal center of China Three Gorges University (license number: SCXK (E) 2017–0012). All rats were acclimated for seven days before the experiment started. Rats were housed (*n* = 4 per cage) at 20 ± 2°C with a relative humidity of 50% ± 10% under a 12-hour light/dark cycle (lights on at 8:00 a.m.), and all rats had free access to food and water. All animal procedures were approved by the Animal Ethics Committee of the Hubei University of Chinese Medicine, Wuhan, China. Rats were randomly assigned to four groups (*n* = 12 per group) using a random number table: control group, model group, preventive electroacupuncture treatment group (EA), and sham electroacupuncture treatment group (sham EA). Rats in the model group, EA group, and sham EA group were injected D-galactose (D-gal) intraperitoneally daily (120 mg/kg/day) for consecutive eight weeks [36, 37]. Rats in the control group received no intervention.

### 2.2. EA Treatment

EA treatment was performed as described in our previous study [32]. Starting on the first day after intraperitoneal injection of D-gal, preventive electroacupuncture treatment was administrated at GV20 and alternating unilateral BL23 to rats in EA and sham EA groups once daily for eight weeks with one rest day every 6 days. GV20 was located at the center of the parietal bone, and the needle was inserted into the epicranial aponeurosis. BL23 was located adjacent to the second lumbar vertebra [38]. During EA treatment, rats were wrapped with tailored and soft cloth materials and at prone position in a conscious state rather than anesthetized. EA was delivered using stainless steel needles (15 mm in length and 0.3 mm in diameter; Beijing Zhongyan Taihe Medical Apparatus Co., Ltd., Beijing, China) and an EA apparatus (HANS-100A, Beijing Huayun Ante Science and Technology Co., Ltd., Beijing, China). At GV20 point, the needle was inserted 15° obliquely to a depth of 2 mm. At BL23, the needle was inserted perpendicularly to a 4–6 mm depth. Based on our previous findings [32, 39], EA at a frequency of 50 Hz showed better neuroprotection effects in A*β*_1-42_-induced rats, so stimulation in a continuous wave at a frequency of 50 Hz in EA group for 20 minutes each day was applied to the pair of acupuncture points (GV20 and alternating unilateral BL23). The intensity (1 mA) was adjusted to induce light muscle contractions. In the sham EA group, only the surfaces of GV20 and BL23 were stimulated, but the needles were inserted without current. Rats in the control group and model group were wrapped in the same way but received no EA treatment.

### 2.3. Morris Water Maze Test

Morris water maze test was performed as described in our previous study [32]. The apparatus (Chengdu Taimeng Technology Co., Ltd., Chengdu, China) consisted of a large circular pool (120 cm in diameter, 50 cm in height) and a MT-200 Morris water maze video-tracking system (Chengdu Taimeng Technology Co., Ltd., Chengdu, China). The data and swimming path were recorded using Watermaze 2.0 software (Chengdu Taimeng Technology Co., Ltd.). The pool was filled with opaque water (24 ± 2°C) to a depth of 40 cm with white milk and divided into four equal quadrants. The circular platform (10 cm in diameter) was placed at the midpoint of the target quadrant and submerged approximately 1.5 cm below the surface of the water. All rats were trained for five consecutive days on standard place navigation trials. Rats were placed into a quadrant facing the wall of the pool on four occasions (every time in different quadrants). The rats were given no more than 120 seconds to find the hidden platform in each trial and the time was recorded as the escape latency. When the rat failed to find the platform within this period, it would be assisted onto the platform and kept staying on the platform for 10 seconds. The rats rested for 30 seconds between trials. For the spatial probe trial (conducted the next day after the place navigation test), the platform was removed from the pool. This test was performed in the same way as the place navigation test. The rats were given 120 seconds to swim in the pool. The time spent in the target quadrant (the quadrant where the hidden platform was during training trials) was recorded.

### 2.4. Western Blot Assay

Six rats from each group were randomly selected for Western blot assay. Lysates were prepared as previously described [32]. Equal amounts of protein from each sample were separated by Tris-glycine SDS-PAGE and transferred onto nitrocellulose membranes (Millipore, Billerica, MA, USA) electrophoretically. After blocking with 5% nonfat dry milk in Tris-buffered saline with 0.1% Tween 20 for one hour at room temperature, the membranes were incubated overnight at 4°C with the following primary antibodies: GSK3*β* (ab93926, Abcam, Inc, USA, at1: 500 dilution), *GSK3β-*pTyr216 (ab75745, Abcam, Inc, USA, at1: 500 dilution), mTOR (20657-1-AP, Proteintech. Wuhan. China, at1: 300 dilution), mTOR-pSer2448 (#5536, Cell Signaling Technology, Danvers, MA, at 1:500 dilution), LC3A/B (#4108, Cell Signaling Technology, Danvers, MA, at 1 : 500 dilution), tau5 (ab80579, Abcam, Inc, USA, at 1 : 500 dilution), PHF-1 (ab184951, Abcam, Inc, USA, at 1 : 1000 dilution), PSD95 (#3450, Cell Signaling Technology, Danvers, MA, at 1:800 dilution), and synapsin-1 (#5297, Cell Signaling Technology, Danvers, MA, at 1 : 1000 dilution). After washes, the membranes were then incubated with the secondary antibodies at 37°C for one hour. A chemiluminescence detection system (ChemiDoc™ XRS+, BIO-RAD, CA, USA) was used to measure the protein signal. All Western blot data were quantified with Image-Pro Plus 6 software (National Institutes of Health, Bethesda, MD, USA). Normalization was performed by blotting the same membranes with GAPDH confirmed by triplicate measurements of the same sample.

### 2.5. Transmission Electron Microscopy (TEM)

Three rats in each group were randomly selected for electron microscopy. Briefly, rats were transcardially perfused with 4% paraformaldehyde and 1% glutaraldehyde in 0.1 M phosphate buffer. Then, approximately 2 mm^3^ brain tissue blocks were immediately cut from the hippocampal CA1 area and were postfixed in 2.5% glutaraldehyde at 4°C for 4 hours and later fixed with 1% osmium tetroxide (2 h, 4°C). The samples were dehydrated in a graded series of acetone (50%, 70%, 90%, and 100%) and embedded in Epon resin at room temperature for 24 hours. Tissue blocks were then cut into 1 *μ*m thick thin sections and mounted on Formvar-coated grids. After uranyl acetate/lead citrate double staining, microtubules in neurons were observed using a transmission electron microscope (Tecnai G^2^ 20 TWIN, FEI, USA). The density of microtubules (measured by the number of microtubules crossed by the line perpendicular to the long axis of the axon as described by Huang et al. [40]) in each group were evaluated and compared.

### 2.6. Golgi Staining

We used an FD Rapid GolgiStain Kit (FD Neurotechnologies, Inc., Columbia, MD, USA) to perform the Golgi staining. Another three rats in each group were randomly selected for Golgi staining. According to the manufacturer's instructions, the cut brain tissues were first incubated in a mixture of A and B solutions from the kit and next stored in dark condition at room temperature. Then, the brain tissues were transferred into solution C from the kit and stored at 4°C for 1 week. Finally, brain tissues were frozen. A cryostat (Leica CM1860, Tokyo, Japan) was used to make coronal sections that are 100 m thick. The sections were incubated in a mixture of D and E solutions from the kit and then dehydrated in a graded series of alcohol (60, 75, 80, 95, and 100% for 5 minutes each), cleared in xylene, and were cover-slipped with neutral gum. Finally, the dendritic spines in the hippocampal CA1 area were viewed using a microscope (Olympus BX53, Tokyo, Japan). Three brain sections in each group were viewed and three randomly selected fields in CA1 area in each section were photographed. The dendritic spine density (determined by the total spine numbers per 10 *μ*m-length dendrites in CA1 area as described by Liu et al. [41] and Cai et al. [42]) was quantified with Image-Pro Plus 6 software (National Institutes of Health, Bethesda, MD, USA). To avoid bias in counting spines using Image-Pro Plus 6 software, spine numbers in each filed were calculated by two investigators in our group to achieve a consensus.

### 2.7. Statistical Analysis

Data were tested for normal distribution using the Kolmogorov–Smirnov test. Normally distributed data were expressed as the mean ± SD and analyzed using GraphPad Prism 7.0 for Windows statistical software (GraphPad Software, California, USA). Statistical significance was determined by one-way ANOVA with a least significant difference test or a one-way ANOVA with post hoc Dunnett's T3. A level of *P* < 0.05 was considered statistically significant.

## 3. Results

### 3.1. Preventive Electroacupuncture Ameliorated Spatial Learning and Memory Deficits in D-Gal-Induced Rats

**Figure 1:**
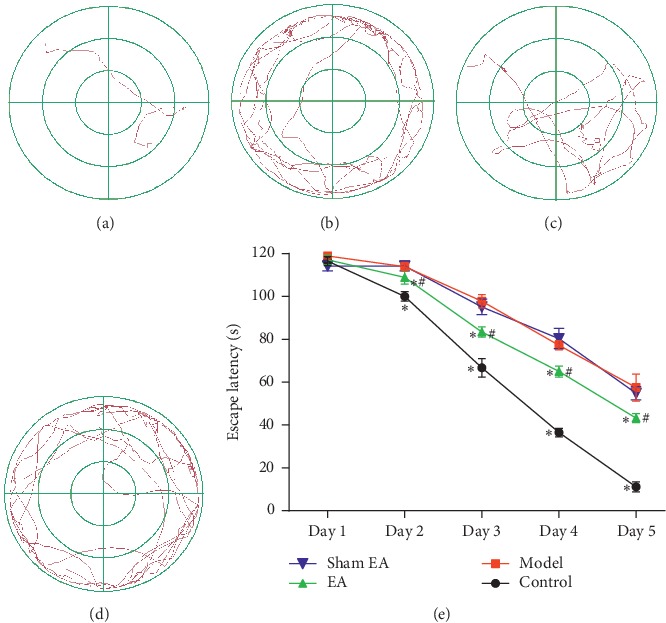
Preventive electroacupuncture improved D-gal-induced spatial memory deficits evaluated by the Morris water maze test. (a–d): The path to find the hidden platform in place navigation trials (a: control, b: model, c: EA, d: sham EA, e: comparison of the escape latency). The data were expressed as mean ± SD (*n* = 12). ^*∗*^*P* < 0.01 vs. model; ^#^*P* < 0.01 vs. sham EA.

**Figure 2:**
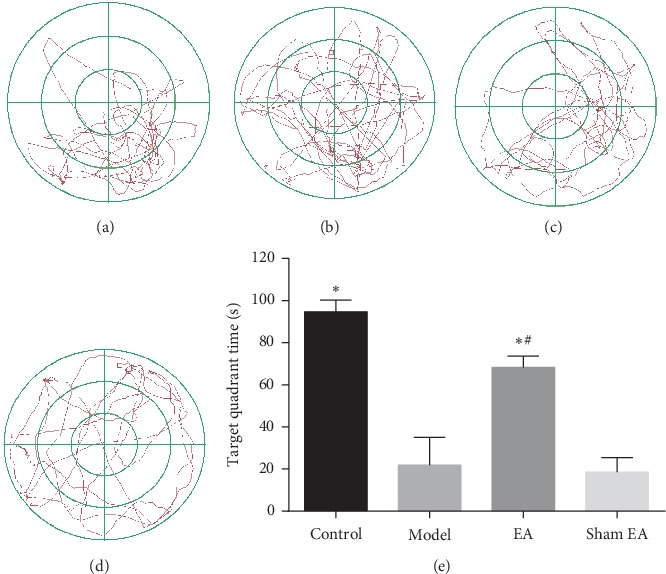
Preventive electroacupuncture attenuated D-gal-induced spatial memory deficits evaluated by the Morris water maze test. (a–d): The path in the target quadrant in 120 seconds after the platform was removed in spatial probe trials: (a) control, (b) model, (c) EA, (d) sham EA, and (e) Comparison of the escape latency. The data were expressed as mean ± SD (*n* = 12). ^*∗*^*P* < 0.01 vs. model; ^#^*P* < 0.01 vs. sham EA.

As shown in Figure 1, the escape latency to find the hidden platform in D-gal rats was significantly increased compared with the control group from day 2 (*P* < 0.01), while preventive EA treatment significantly decreased the escape latency (*P* < 0.01) when compared with the control and sham group (*P* < 0.01) (Figure 1(e)). The memory impairment was also detected by removing the hidden platform. As shown in Figure 2, the time spent in the target quadrant of rats in the model group was decreased compared with the control group (*P* < 0.01), and preventive EA treatment significantly increased the target quadrant time (*P* < 0.01).

### 3.2. Preventive Electroacupuncture Increased Dendritic Spine Density in the Hippocampal CA1 Area in D-Gal-Induced Rats

**Figure 3:**
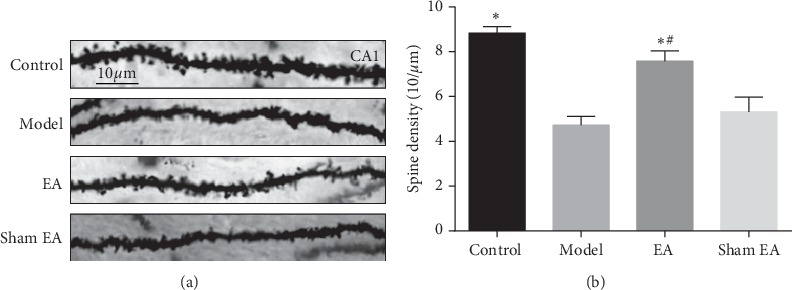
The dendritic spine density in the hippocampal CA1 area in D-gal-induced rats. (a) Representative images of dendritic spine density in the pyramidal cell layer of the hippocampal CA1 area in each group (Golgi staining, scale bar = 10 *μ*m). (b) The quantification of spine density from randomly selected dendritic segments of neurons. The data were expressed as mean ± SD (*n* = 3). ^*∗*^*P* < 0.01 vs. model; ^#^*P* < 0.01 vs. sham EA.

To evaluate the effects of preventive EA treatment on synaptic morphology, dendritic spine density in the hippocampal neurons was analyzed by Golgi staining. As illustrated in Figure 3(a) and 3(b), the density of dendritic spines in the model group was significantly decreased compared with the control (*P* < 0.01). Preventive EA treatment ameliorated the loss of dendritic spines (^*∗*^*P* < 0.01 vs. model; ^#^*P* < 0.01 vs. sham EA).

### 3.3. Preventive Electroacupuncture Ameliorated Microtubule Impairment in Hippocampal CA1 Area in D-Gal-Induced Rats

**Figure 4:**
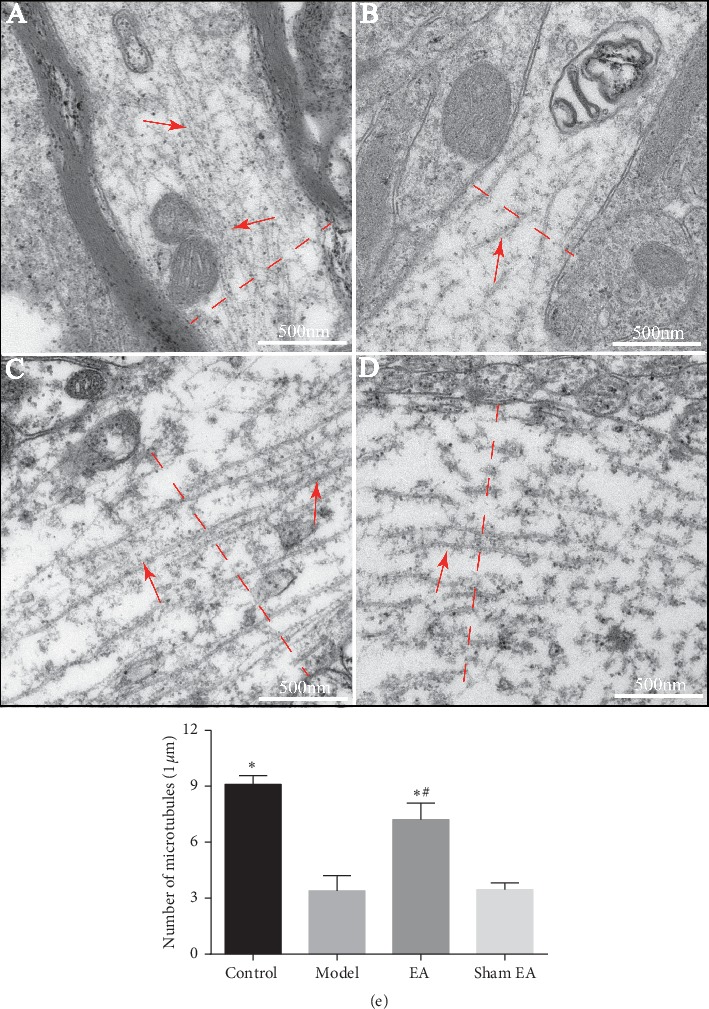
Representative microtubules in axon in the hippocampal CA1 area in D-gal-induced rats (transmission electron microscopy, scale bar = 500 nm, *n* = 3). Red arrow: microtubules. (a) Control, (b) model, (c) EA, and (d) sham EA. The microtubule density was calculated by scoring the number of microtubules crossed by the line perpendicular to the long axis of the axon (red dashed line). (e) The quantification of microtubule density from randomly selected fields. The data were expressed as mean ± SD (*n* = 3). ^*∗*^*P* < 0.01 vs. model; ^#^*P* < 0.01 vs. sham EA.

Results from electron microscopy showed that the microtubules in neurons in the hippocampal CA1 area in D-gal-induced rats were obviously fractured and sparse when compared with the control group. Compared with the model group and sham EA group, microtubules in the EA group were longer and less fractured (Figures 4(a)–4(d)). As shown in Figure 4(e), the density of microtubules in the model group was significantly decreased compared with the control (*P* < 0.01). Preventive EA treatment significantly increased the density of microtubules (^*∗*^*P* < 0.01 vs. model; ^#^*P* < 0.01 vs. sham EA).

### 3.4. Preventive Electroacupuncture Decreased Tau Hyperphosphorylation and Upregulated the Levels of PSD95 and SYN

**Figure 5:**
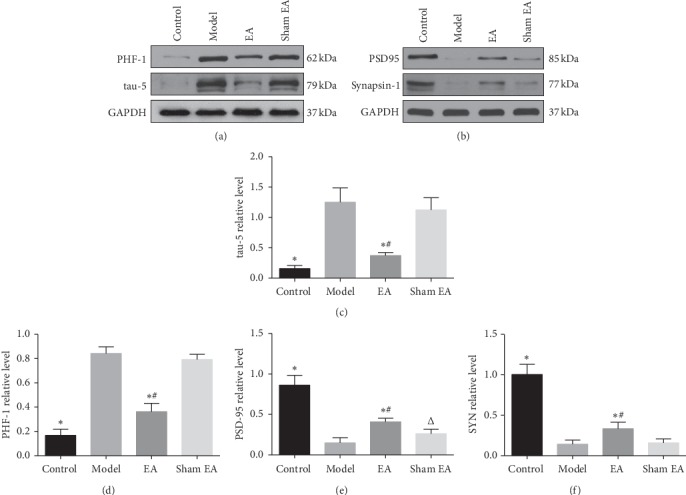
Preventive EA decreased D-gal-induced tau hyperphosphorylation levels and preserved synapse-associated proteins. The protein levels of tau-5, PHF-1, PSD95, and synapsin-1 in the hippocampus were detected using western blotting (a, b). Comparison of the relative expression of tau-5, PHF-1, PSD95, and SYN in the hippocampus (c,d,e,f). GAPDH was used as a loading control. The data were expressed as mean ± SD (*n* = 6). ^*∗*^*P* < 0.01 vs. model; ^#^*P* < 0.01 vs. sham EA.

Tau phosphorylation is one of the two hallmarks of AD. D-gal was reported to induce tau hyperphosphorylation in rats [43, 44] and impact synaptic plasticity [45], so the expression levels of PHF-1 recognizing tau Ser396/404 were evaluated by Western blot. As shown in Figure 5(d), preventive EA prevented and rescued the tau hyperphosphorylation (*P* < 0.01). Moreover, preventive EA decreased the immunoreactivity of Tau-5 epitope recognizing the total tau (Figure 5(c), *P* < 0.01). Synapse-associated proteins such as PSD95 in the postsynapse and synapsin-1 in the presynapse impacts synaptic function and are involved in learning and memory [46, 47]. Therefore, the expression levels of PSD95 and synapsin-1 were investigated using western blotting. We found that D-gal decreased the levels of PSD95 and synapsin-1 in the hippocampus (Figures 5(b), 5(e) and 5(f)), while preventive EA increased the expression levels of PSD95 and synapsin-1 (*P* < 0.01). These results suggest that preventive EA effectively decreased D-gal-induced tau hyperphosphorylation levels and rescued synapse impairments.

### 3.5. Preventive Electroacupuncture Inhibited GSK3*β*/mTOR Signaling Activity in D-Gal-Induced Rats

**Figure 6:**
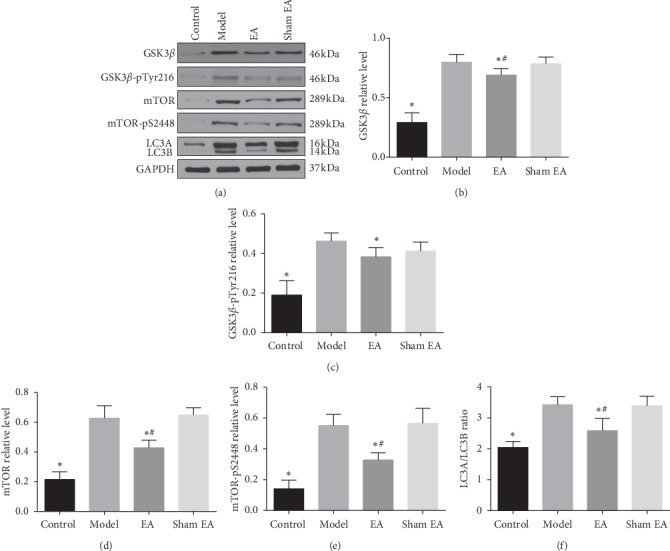
Preventive EA inhibited GSK3*β*/mTOR signaling pathway in D-gal-induced aging rats. The protein levels of GSK3*β*, GSK3*β*-pTyr216, mTOR, mTOR-pSer2448, LC3A, LC3B in the hippocampus were detected using western blotting (a). Comparison of the relative level of GSK3*β*, GSK3*β*-pTyr216, mTOR, mTOR-pSer2448, and LC3A/LC3B ratio in the hippocampus (b,c,d,e,f). GAPDH was used as a loading control. The data were expressed as mean ± SD (*n* = 6). ^*∗*^*P* < 0.01 vs. model; ^#^*P* < 0.01vs. sham EA.

As presented in Figure 6, the levels of GSK3*β*, GSK3*β*-pTyr216, mTOR, mTOR-pSer2448, and LC3A/LC3B ratio in hippocampus were increased in the model group compared with that of control group (*P* < 0.01). No statistical differences were observed between the model and the sham EA group (*P* < 0.05). Compared with the model group, EA treatment significantly decreased the levels of GSK3*β*, GSK3*β*-pTyr216, mTOR, mTOR-pSer2448, and LC3A/LC3B ratio (*P* < 0.01). These results imply that preventive EA treatment could inhibit GSK3*β*/mTOR signaling activity, leading to a reduction of tau hyperphosphorylation and promoted autophagic activity.

## 4. Discussion

AD has become the most common cause of dementia, affecting a growing number of aging populations [3]. The prevalence of AD is increasing with the acceleration of global aging [48]. So it is critical and urgent to find an effective therapy for this devastating disorder. Intracellular NFTs accumulation as a result of tau hyperphosphorylation and aggregation is the early pathology in AD [10], and NFTs deposition can even be found in aging brains without obvious memory deficits [11]. Therefore, prevention of neurofibrillary tangles accumulation as a result of tau hyperphosphorylation [49] and autophagy degradation deficits [50] may be a promising efficient intervention strategy for AD. In the present study, we investigated whether preventive EA treatment during aging could attenuate memory deficits in consequence of tau hyperphosphorylation. We found that the levels of hyperphosphorylated tau in the hippocampus in aging rats were higher than that of control, as indicated by elevated PHF-1 and tau-5. In addition, more fractured and sparser microtubules and more loss of dendritic spines were observed in the hippocampal CA1 area. Preventive EA treatment finally ameliorated synapse and microtubule morphological damage as well as upregulated synapse-associated proteins PSD95 and SYN levels, which were critical for synapse transmission and memory [46, 47]. Then we measured GSK3*β*/mTOR signaling activity that was associated with synaptic plasticity [51], tau hyperphosphorylation [19], and autophagic clearance of hyperphosphorylated tau [52]. As expected, a remarkable inhibition of GSK3*β*/mTOR signaling pathway by preventive EA was observed. These data suggested that inhibition on GSK3*β*/mTOR signaling pathway by preventive EA could participate in rescuing synapse injuries and memory deficits in D-gal-induced aging rats.

It has been reported that acupuncture can yield protective effects on AD via various pathways such as attenuating neuroinflammation, inhibiting A*β* protein deposition and tau hyperphosphorylation, enhancing glucose brain metabolism, reducing oxidative stress and inhibiting neuronal apoptosis [53, 54]. However, there has been no investigation on the effects of acupuncture on the dendritic spines and microtubules in AD-like pathological rodents, especially in aging animals. In addition, current studies paid more attention to studying the effects of acupuncture in AD mice with existing typical pathological hallmarks, and few investigated whether preventive EA could prevent or alleviate synaptic failure and memory impairment during aging that has not evolved into AD but shared similar characteristics such as neuroinflammation, oxidative stress, and epigenetic changes [55]. Since there has been no well recognized disease-modifying treatment for AD worldwide, it makes sense that preventive strategies based on targeting risk factors like aging should be considered. In the present study, acupoints GV20 and BL23 belonging to Governor Vessel and Bladder Meridian of Foot-Taiyang were chosen, respectively, for the deficiency of kidney and Governor Vessel occlusion as a result of qi stagnation and blood stasis contributing to the pathogenesis of AD according to Chinese acupuncture theory. Our previous data mining analysis also indicated that GV20-BL23 acupoints served as one of the most dominant acupoint combinations for the treatment of AD [56]. In addition, neuroimaging studies revealed that the therapeutic effects of acupuncture at GV20-BL23 acupoints are associated with inducing specific patterns of neural responses involved in limbic/paralimbic regions and frontal lobes [57]. As expected, we observed that preventive EA treatment at GV20-BL23 acupoints attenuated synapse and microtubule morphological injuries and increased the levels of synapse-associated proteins in D-gal-induced aging rats.

Several reviews have discussed the intrinsical interweaving between Alzheimer's disease and aging in molecular, cellular, and systemic aspects [55, 58]. Aging in humans is relevant to decreased levels of synaptic proteins related to synaptic structural plasticity [59], and aging-related spatial learning impairments correlate with the hippocampal synapse failure [60–64]. Synaptic plasticity is determined by normal synapse structure and functional synaptic proteins. The dendritic spines are the main postsynaptic components of synapses and are primary structures for learning, memory, and cognition [65]. Specially, the new dendritic spines can, in part mediate the long-term memory formation to strengthen a particular neural circuit [66]. Compelling evidence supports the significant role of PSD95 and SYN in promoting synapse formation and reconstruction and coherence of presynaptic and postsynaptic actions are necessary for synaptic transmission [67–69]. Presynaptic synapsin-1 is a marker vesicle protein of synaptic density, and it is involved in the release of various neurotransmitters [67]. PSD95 is in the postsynaptic density, and its interaction with N-methyl-D- aspartate (NMDA) receptors engages in the memory storage process [70]. Moreover, existing evidence already demonstrated a decline of PSD95 in aging [71]. A downregulation of PSD95 may be the beginning of the synaptic plasticity damage prior to the synaptic loss in the pathology of AD [72]. In addition, increased levels of synaptic proteins are associated with superior learning and memory in aged animals [73]. Our results in the present study showed that preventive EA during aging increased expressions of PSD95 and SYN and attenuated memory impairment, which was consistent with the findings of Dong et al. [74] and our previous study [75].

As discussed in previous reviews [19, 51, 76], GSK3*β* functions in the assembly and disassembly of synapses determining synaptic plasticity. Except for the wide-recognized role in the regulation of tau phosphorylation, GSK3*β* activity also participated in proper synaptic development [77, 78] and neurotransmission [79, 80]. Knockout of the GSK3*β* in the hippocampal and cortical neurons results in a reduction of spine density, loss of persistent spines, and a reduction of the ability of new spines to stabilize, together with a decrease of AMPA-receptor-mediated miniature excitatory postsynaptic currents [81]. A recent study also reported that matrix metalloproteinase-9 can serve as a mediator of GSK3*β*-induced the maturation of dendritic spines [82]. Conversely, overexpression of GSK3*β* alters dendritic branching and causes a decreased number of the functional synapses of dentate gyrus granule neurons [83]. It is noted that dysregulation of mTOR is implicated in tau hyperphosphorylation and degradation [52] and memory loss [84]. Inhibition of mTOR may have the potential to treat age-related synaptic dysfunction and cognitive deficits [85, 86]. LC3 participates in the biogenesis and maturation of the autophagosome. Conversion from a nonlipidated form (LC3 I) to a phosphatidylethanolamine-conjugated form (LC3 II) is necessary for forming completed and functional autophagosomes and elevated LC3 II/LC3 I ratio indicates an enhanced autophagy activity [87]. Avrahami et al. recently demonstrated that GSK3*β* acted as a “positive regulator” on the mTOR signaling [25]. They found that the inhibition of GSK3*β* restored lysosomal acidification that, in turn, prompted the clearance of A*β* plaque load via reactivation of mTOR. It was also proved that GSK3*β* activated mTOR by targeting eukaryotic elongation factor-2 [88]. Thus, the inhibition of GSK3*β*/mTOR signaling pathway could be a promising target of drug discovery for AD [89]. Recent evidence demonstrated that electroacupuncture can induce neuroprotective effects in AD and Parkinson's disease animals by the regulation of mTOR-independent autophagic clearance [90, 91]. A recent study also found that early preventive EA decreased the phosphorylation levels of tau at Ser202/Thr205 and Thr231 sites and ameliorated memory deficits in SAMP8 mouse, another aging mouse model. But the possible underlying molecular pathways and morphological evidence were not investigated [92].

Herein, we observed that preventive EA rescued structural and functional synaptic plasticity impairments and memory deficits, probably by inactivation of GSK3*β*/mTOR signaling.

However, several cautions should be noted in this study. Long-term potentiation (LTP) and long-term depression (LTD), another vital indicators measuring synaptic plasticity [93], were not detected by the electrophysiology method, while we also doubt that whether it is rational to measure the synaptic transmission using the electrophysiology method in vitro to investigate the acupuncture effects since the physiological responses and effects induced by acupuncture occur in vivo. Whether the activity of neurons extracted from the living brain, though cultured alive in vitro, is induced by acupuncture remains controversial in the acupuncture field. Second, we only explored the effects of preventive EA in D-gal-induced aging rats; the effect of preventive EA should be measured in other aging models, such as SAMP8 mice with AD traits. Third, it could be more convincing to additionally investigate the tau phosphorylation and aggregation using immunohistochemistry method or double-labeling immunofluorescence with autophagosome markers to present more visualized morphological changes. The autophagic clearance processing of pathological tau proteins will be investigated in future studies.

In conclusion, here we provide evidence supporting the finding that preventive EA at GV20-BL23 acupoints during aging process can attenuate the dendritic spine loss, rescue neuronal microtubule injury, and ameliorate learning and memory deficits in D-gal-induced aging rats. This neuroprotective effects could be associated with the inhibition of GSK3*β*/mTOR pathway involved in tau phosphorylation and autophagic degradation. Our results could provide new insight into the exploration of prevention strategies for AD.

## Data Availability

The data used to support the findings of this study are available from the corresponding author upon request.
